# Lowered blood pressure targets identify new, uncontrolled hypertensive cases: patient characteristics and implications for services in Thailand

**DOI:** 10.1186/s12913-020-05719-z

**Published:** 2020-09-14

**Authors:** Naphassanan Charoensab, Kanokporn Pinyopornpanish, Phaviga Thangsuk, Wichuda Jiraporncharoen, Chaisiri Angkurawaranon

**Affiliations:** 1 Department of Family Medicine, Faculty of Medicine, Chiang Mai University, 110 Intawarorot Rd, Sriphum, Muang, Chiang Mai, 50200 Thailand; 2 Chiangrai Prachanukroh Hospital, Chiang Rai, Thailand

**Keywords:** Hypertension, Clinical management, Thailand, Clinical practice guidelines, blood pressure control

## Abstract

**Background:**

According to the new hypertension treatment guidelines blood pressure (BP) readings need to be kept below or equal to 130/80 mmHg in patients aged less than 65 years old. This study shows the change in proportion of identified cases of uncontrolled blood pressure in light of these changes.

**Methods:**

The data was collected from 248 hypertensive patients who had visited an outpatient clinic at the Department of Family Medicine, Faculty of Medicine, Chiang Mai University, Thailand. Patients were classified into three groups: The 3 groups were: 1) controlled BP group (BP is 130/80 mmHg or lower); 2) newly identified uncontrolled group (BP between 130/80 mmHg and 140/90 mmHg) and 3) existing uncontrolled group (BP higher than 140/90 mmHg). Health behaviors, past history related to hypertensive disease and current pharmacological treatments were compared.

**Results:**

Of the total 248 patients, 56% were female and the mean age was 58.8 (sd 5.99) years old. Following application of the new guidelines, the group designated as uncontrolled increased from 21.7 to 74.2%, an additional 52.4% due to new BP targets. Higher BMI was associated with uncontrolled HT (*p* = 0.043). While the average number of medication taken was similar across the three groups, it was poor medication adherence (*p* < 0.013) which was associated with the uncontrolled disease.

**Conclusions:**

Lower BP targets will increase the number of identified hypertensive patients. While intensifying pharmacological treatment may be considered, our study suggests that two behavioral factors should not be overlooked. Weight reduction and enhancement of medication adherence remains an important mainstream treatment strategy.

## Background

Hypertension (HT) is a chronic disease which is of a great concern as it is one of the cardiovascular risk factors and leads to the disease burden on cardiovascular diseases (CVD) morbidity and mortality [1]. The World Health Organization (WHO) has identified that the overall prevalence of hypertension in adults is around one out of three. The high prevalence of hypertension is globally consistent and continues to rise [2]. According to the latest Thai National Health Examination Survey conducted in 2015, one out of four Thai people have hypertension. Among those cases of hypertension, less than one out of three had their blood pressure under control [3]. Uncontrolled blood pressure (BP) leads to CVD, retinopathy, kidney disease and cerebrovascular disease [4]. Both systolic and diastolic hypertension independently influences the risk of adverse cardiovascular disease [5]. Nevertheless, regular control of blood pressure (BP) in mild and moderate hypertension group can significantly reduce 25% of major cardiovascular (CV) events and 27% of all-cause mortality [6].

Guidelines from the 2018 American College of Cardiology (ACC) and the American Heart Association (AHA) recommends a lower blood pressure goal for treatment strategies in hypertension, the blood pressure goal is decreased from 140/90 mmHg to 130/80 mmHg in every patient aged less than 65 years old. Following the Systolic Blood Pressure Intervention Trial (SPRINT) [7], it was reported that more intensive BP-lowering treatment was associated with a 25% reduction in major CV events and a 27% reduction in all cause death, both statistically significant [6]. This outcome provides strong support for the beneficial effects of more intensive BP-lowering treatment strategies.

The Thai Guidelines on The Treatment of Hypertension 2019 [8] was launched officially in August 2019. The BP target has been adopted from the 2018 European Society of Cardiology/European Society of Hypertension (ESC/ESH) Guidelines as 130/80 mmHg and lowered for people aged 18–65 years old without consideration of any co-morbidities. However, lowering target BP would result in greater numbers of patients with identified uncontrolled blood pressure. A new patient population will also emerged from reclassification of BP control, as part of patients who were considered as controlled will now be reclassified as uncontrolled. The expected increase number of patients requiring intensive BP-lowering treatment strategies, would lead to a greater requirement for more treatment resources.

This study aims to determine the prevalence of newly identified uncontrolled patients in accordance with the new BP goals. The patient characteristics, background health behaviors and pharmacologic treatment were investigated resulting in comparisons between three groups consisting of 1) BP controlled group, 2) a group in which the BP had already been acknowledged as not controlled and 3) a new group in which the BP was identified as uncontrolled using the new stricter guidelines. Understanding the patient characteristics and potential differences between the three groups will be beneficial for further interventions and health care service planning such as reducing modifiable risk factors and guide clinical practices in countries such as Thailand where new BP targets are being adopted.

## Methods

We conducted a cross-sectional study of patients with hypertension who had visited the out-patient clinic of the Department of Family Medicine, Faculty of Medicine, Chiang Mai University. Sample size calculation was based on a prior population based study from Thailand that the prevalence of controlled hypertension was estimated at approximately 20% [9]. Allowing an alpha error of 5%, a power of 80% and a precision of 5%, it was estimated that at least 246 participants was needed. We sequentially recruited and enrolled two hundred forty-eight patients aged between 18 and 65 years old, diagnosed as having hypertension for at least 3 months. Trained research assistants conducted a face-to-face interview to administer the survey questionnaire and reviewed secondary data from electronic medical records (additional file 1). Participants gave their written informed consent prior to each interview. The study was approved by the Ethics Committee from Chiang Mai University (No. 6398/2562).

The interviews consisted of assessment of current health behaviors, past history related to hypertensive disease and current pharmacological treatments. Health behaviors assessed included taking alcohol consumption, and smoking in past 3 months, a history of adequate exercise (moderate intensity aerobic exercise more than 150 min per week in the past 3 months), sodium consumption (assessed by self-report of adding flavoring agents in at least 1 meal per day). These were assessed by yes or no answers. A history of illness related to hypertensive disease include family history of HT, family history of CVD or IHD, co-morbidities such as diabetes and dyslipidemia. For pharmacological therapy related to HT treatment, information was collected on the number of years diagnosed with HT, duration (years) for taking anti-hypertensive drugs, the class of drugs currently taken, the types of anti-hypertensive drugs currently taken per day, the number of times taking anti-hypertensive drugs, combined drugs currently used, experience of side effects from anti-hypertensive drug. Research assistants directly measured height and weight by using the same stadiometer and electronic scale throughout the study. Self-reported medication adherence using the Thai version of the four-item Morisky Green Levine (MGL) Medication Adherence Scale [10] were also derived from the interview. The author (CA) received the original MGL questionnaire from the Morisky Medication Adherence Research, LLC to use and translate. The translation of the questionnaire followed the WHO suggested standard procedure consisting of forward and back-translation by expert, pretesting and cognitive interviewing before developing the final version [11]. The 4-item questionnaire assessed whether participants have i) ever forgotten to take their medication, ii) at times been careless about taking medicine, 3) sometimes stopped taking medication when they felt better or 4) sometimes stopped taking medication when they felt worse. The participant had to answer “No” to all four items to be considered as having high medication adherence.

After the patient has rested for at least 30 min, we measured each person’s blood pressure twice, 15-min apart, using an automatic sphygmomanometer. The average value of the two readings was used for analysis. Based on the BP readings, patients were categorized into three groups using the new goals for BP control described in the Thai Hypertensive Guidelines 2019 [8]. The Thai Hypertension Guideline follows the 2018 ESC/ESH which states that in patients less than 65 years of age their BP should be kept below or equal to 130/80 mmHg regardless of any co-morbidity. The 3 groups were: 1) controlled BP group (BP is 130/80 mmHg or lower); 2) newly identified uncontrolled group (BP between 130/80 mmHg and 140/90 mmHg) and 3) existing uncontrolled group (BP higher than 140/90 mmHg).

### Statistical analysis

The data was analyzed using STATA 15.1. Descriptive statistics were used to summarized patient characteristics, past medical history, current behavior and treatment. Chi – square or Fischer’s exact test and analysis of variance (ANOVA) were used to compare qualitative and quantitative variables respectively in order examine potential differences between the three groups of patients.

## Results

Out of the 248 hypertensive patients, 141 were female (56%) and the mean age was 58.8 (sd 5.99) years old. The patients had been diagnosed with hypertension for 8.1 (sd 4.69) years. According to the new Thai Hypertensive Guidelines 2019, the controlled group was 25.8% of the sample, the new uncontrolled group was 52.4% and the pre-guidelines uncontrolled group was 21.7%.

As shown in Table 1, the only differences in demographics between these three groups was the average body mass index (BMI). The BMI in the controlled group, new uncontrolled group and pre-guidelines uncontrolled group were 26.1 ± 3.86 kg/mg^2^, 27.8 ± 4.52 kg/mg^2^, and 27.4 ± 4.75 kg/mg^2^, respectively (*p* = 0.043). While all BMI of all these groups was considered high, the BMI in the controlled BP group was the lowest. In all three groups, there was no evidence for any differences in co-morbidities and family history of cardiovascular diseases (Table 2**).**

**Table 1:** Demographic data

	Controlled Blood Pressure (*N* = 64)	Post-Guidelines Uncontrolled Blood Pressure (*N* = 130)	Pre-Guidelines Uncontrolled Blood Pressure (*N* = 54)	*P* value
Female gender, *n*(col %)	41 (64.06)	67 (51.54)	33 (61.11)	0.197
Age (years), Mean (SD)	59.7 (5.17)	58.2 (6.05)	58.7 (6.68)	0.264
BMI (kg/m^2^), Mean (SD)	26.1 (3.86)	27.8 (4.52)	27.4 (4.75)	0.043
Marital status, *n*(col %)				0.229
Single/Divorced	13 (20.31)	16 (12.31)	11 (20.37)	
Married	51 (79.69)	114 (87.69)	43 (79.63)	
Nationality, *n*(col %)				0.236*
Thai	63 (98.44)	130 (100)	54 (100)	
Other	1 (1.56)	0	0	
Religion, *n*(col %)				0.205
Buddhism	61 (95.31)	126 (96.92)	49 (90.74)	
Other	3 (4.69)	4 (3.08)	5 (9.26)	
Educational level, *n*(col %)				0.609
Primary school	17 (26.56)	33 (25.38)	13 (24.07)	
Secondary school or voluntary school	13 (20.31)	40 (30.77)	14 (25.93)	
Bachelor’s degree or higher	34 (53.13)	57 (43.85)	27 (50.00)	
Having health insurance, *n*(col %)	60 (93.75)	109 (83.85)	52 (96.29)	0.057
Involvement of caregiver in medication management, *n*(col %)	5 (7.81)	5 (3.85)	0	0.098
Having an occupation, *n*(col %)	52 (81.25)	105 (80.77)	41 (75.93)	0.326

* Fischer’s exact test

**Table 2:** History of illness related to hypertensive disease

	Controlled Blood Pressure (*N* = 64)	Post-Guidelines Uncontrolled Blood Pressure (*N* = 130)	Pre-Guidelines Uncontrolled Blood Pressure (*N* = 54)	*P* value
Family history of hypertension, n(col %)	48 (75)	80 (61.53)	37 (68.52)	0.188
Family history of CVD or IHD, n(col %)	18 (28.13)	27 (20.77)	12 (22.22)	0.513
Presence of co-morbidity				
• Type 2 Diabetic mellitus, *n*(col %)	13 (20.31)	31 (23.85)	9 (16.67)	0.541
• Dyslipidemia, n (col %)	54 (84.38)	112 (86.15)	47 (87.04)	0.911
• Coronary heart disease, *n*(col %)	2 (3.13)	2 (1.54)	1 (1.85)	0.757
• Cerebrovascular disease, *n*(col %)	1 (1.56)	5 (3.85)	2 (3.7)	0.681
• Obstructive sleep apnea, *n*(col %)	3 (4.69)	7 (5.38)	4 (7.41)	0.802
• Hyperthyroid, *n*(col %)	2 (3.13)	1 (0.77)	0	0.242
• Chronic kidney disease, *n*(col %)	9 (14.06)	12 (9.23)	6 (11.11)	0.596

*CVD* cardiovascular disease, *ED* emergency department, *IHD* ischemic heart disease, *HTN* hypertension

In regard to behavioral aspects, Table 3 showed that all the three groups had similarities. The percentages of patients consuming alcohol in the last three months was around 20 % and less than 5 % smoked. Around 60 % were able to do adequate exercise. In the assessment of pharmacological therapy, the dosing and regimen in anti-hypertensive treatment were also similar. There were no significant differences the classes of anti-hypertensive drug taken, the number of medications currently taken per day, number of times taking anti-hypertensive drugs, or the history of experience of side effects from anti-hypertensive drugs (Table 4**)**. However, high medication adherence was statistically significantly different and was highest in the BP control (*p* = 0.013).

**Table 3:** Health Behaviors

	Controlled Blood Pressure (*N* = 64)	Post-Guidelines Uncontrolled Blood Pressure (*N* = 130)	Pre-Guidelines Uncontrolled Blood Pressure (*N* = 54)	*P* value
History of alcohol consumption in past 3 months, *n*(col %)	12 (18.75)	40 (30.77)	11 (20.37)	0.123
History of smoking in past 3 months, *n*(col %)	3 (4.69)	2 (1.54)	2 (3.70)	0.418
Adequate exercise, *n*(col %)	40 (62.50)	90 (69.23)	37 (68.52)	0.629
Dietary sodium restriction, *n*(col. %)				0.869
Restricted	25 (39.06)	50 (38.46)	23 (42.59)	
Non-restricted (add > = 1 meal/day)	39 (60.94)	80 (61.54)	31 (57.41)

**Table 4:** Pharmacological Therapy

	Controlled Blood Pressure (*N* = 64)	Post-Guidelines Uncontrolled Blood Pressure (*N* = 130)	Pre-Guidelines Uncontrolled Blood Pressure (*N* = 54)	*P* value
Years diagnosed with hypertension (year), Mean (SD)	8.1 (3.68)	8.5 (4.99)	7.3 (4.93)	0.320
Years taking anti-hypertensive drugs (year), Mean (SD)	8 (3.78)	8.4 (4.97)	7.3 (4.93)	0.325
Class of current medications taken per day, Mean (SD)	3.3 (1.62)	3.0 (1.60)	2.7 (1.38)	0.166
Types of anti-hypertensive drugs taken per day,				
Mean (SD)	1.4 (0.64)	1.4 (0.58)	1.4 (0.56)	0.775
Median (IQR)	1 (1–2)	1 (1–2)	1 (1–2)	0.861*
Number of times taking anti-hypertensive drugs,				
Mean (SD)	1.8 (0.46)	1.7 (0.52)	1.7 (0.48)	0.422
Median (IQR)	2 (2–2)	2 (1–2)	2 (1–2)	0.414*
Types of current anti-hypertensive drug				
• Calcium channel blockers, n(col %)	36 (56.25)	74 (56.92)	33 (61.11)	0.842
• ACEIs, *n*(col. %)	11 (17.19)	10 (7.69)	5 (9.26)	0.121
• ARBs, *n*(col %)	26 (40.63)	70 (53.85)	25 (46.30)	0.205
• Thiazide type, *n*(col %)	3 (4.69)	7 (5.38)	4 (7.41)	0.802
• Beta blockers, *n*(col %)	7 (10.94)	13 (10.00)	2 (3.70)	0.312
• Other, *n*(col %)	2 (3.13)	0	1 (1.85)	0.514
Combined drugs use, *n*(col. %)	9 (14.06)	13 (10.00)	6 (11.11)	0.702
Experience of side effects from anti-hypertensive drugs, *n*(col %)	6 (9.38)	11 (8.46)	8 (14.81)	0.418
High medication adherence, *n*(col %)	32 (50.00)	42 (32.31)	14 (25.93)	0.013

*Kruskal-Wallis rank test

## Discussion

This study showed tremendous increase in the identified prevalence of uncontrolled BP patients from 21.7 to 74.2% based on the new BP criteria. In the newly identified uncontrolled BP group, there are significant differences in modifiable risk factors compared to the controlled BP group, specifically BMI and medication adherence.

The prevalence of overall uncontrolled patients was increased by 52.4% (the post-guidelines uncontrolled group). A prior study conducted in the two countries, United States and China, give similar results to our study [12–14]. These studies collected data by observational assessment of nationally representative data from 2013 to 2017 in patients aged between 45 and 75 years old. In the US it was 54.4% in the new uncontrolled group and in China was 76.2% based on the 2018 ACC/AHA guidelines.

Higher BMI is associated with hypertension [15, 16]. As our study suggested that BMI was high in all three groups and significantly higher in the two uncontrolled group, strategies in weight and BMI management should be an area of focus. Literature suggest that non-pharmacological strategies can be effective in weight management. The strategies include drinking eight glasses of water every day which has been shown to will increase body metabolism up to 24–30% [17], dietary carbohydrate restriction (such as rice, starch and sugar) [18–21], aerobic exercise for 30 min every day, 4 times a week (such as jogging, cycling robe jumping and aerobic dance) [22] and high fiber dietary consumption [23]. There was an evidence that harmful alcohol consumption is also associated with increase BMI [24]. Increased energy intake with alcohol use can promote an excess of energy and ultimately weight gain [25]. Many patients need advising that 1 g of alcohol provides 7.1 kcal. Giving advice on the appropriate amount of alcohol might be important for further advice for drinkers, who were about 20–30% of our study population.

In regard to pharmacological factors, interestingly, the average number of the anti-hypertensive medication taken was similar in all groups, but it was medical adherence that was significantly higher among the controlled group. It is a potential cause for concern. Our data suggest that even if pharmacological treatment were to be intensify, there might not be a direct benefit due to poor adherence. A systematic review shows that adherence to prescribed medication leads to significantly lower blood pressure and lower mortality compared with suboptimal adherence [26]. Non-adherence such as stopping taking the medication without permission or discontinuing taking medication, is a common reason for treatment failure, treatment resistance [26] and lack of the advantages including lower incidence of CVD and cerebrovascular disease [27]. Therefore, evaluation of medication adherence is necessary, especially in uncontrolled disease patients.

In low adherence patients, there are many ways to improve medication adherence such as exploring and addressing the barriers to medication adherence for the patient, finding common ground and improving the patient’s health literacy. In addition self-monitoring of blood pressure will help improve the awareness of patient and encourage good self-care [28]. Cognitive behavioral therapy such as motivational interviews conducted with difficult patients and patient with psychosocial conditions can also help when it comes to medication adherence. Furthermore, the use of combination pills for reducing the number of pills to take can improve adherence [29, 30]. Once high adherence is achieved, an adjustment of medication should be the next step [31]. Anti-hypertensive drug therapy can involve at least three types of medication with all at optimal dose or even four [6].

Base on the results, we can expect an increase workload and burden of uncontrol hypertension due to new treatment targets. It has been estimated that less than 1% of cases with hypertension in Thailand are on lifestyle management alone [32] as access to essential antihypertensive medication has already been included as part the Thailand’s universal health coverage scheme since 2011 [33]. However, more support as well as reorientation of the service systems is likely required [34]. For example, introduction of fixed-dose combination (FDC) pills might improve medication adherence which could then improving BP control [35]. However, FDC pills are not yet on the essential drug list in government hospitals in Thailand due to its high cost. In addition, patients may need more contact time with providers to discuss about lifestyle and behavior modifications. Task shifting and delegation of tasks to a multidisciplinary team will likely be needed [36]. Evidence from Thailand also suggest that a more patient-centered approach that stresses holistic and continuity of care may also help improve hypertension control [37] .

There were some limitations of this study. Firstly, using a single site may limit the generalizability of results of other settings. Our study is limited to patients who are less than 65 years of age. The systolic BP should be targeted at between 130 and 140 mmHg, and diastolic BP to less than 80 mmHg for patients ages 65 and older [6]. Thus, our results may overestimate the true burden due to reclassification as a high proportion of patients are already age 65 or older. Using clinic blood pressure could slightly overestimated the number of patients with uncontrolled HT as some patients may have white coat hypertension [38]. However, these clinic-based readings are used in clinical practice and this misclassification will only cause dilution in associations. Some of our measures of patient behavior, particularly salt consumption was very crude. However, this is a commonly used proxy by the Ministry of Public Health in Thailand [39]. Causality cannot be assumed due to the nature of the cross-sectional study design. In addition, this study was not designed to study interventions, further studies looking at non-pharmacological interventions and studies that target improved medication compliance are needed in the Thai context.

## Conclusions

Despite its limitation, the study gave some insights which has clinical implications. Adopting guidelines with lower BP targets such as the new Thai Hypertensive Guidelines 2019 will increase the number of identified hypertensive patients which will increase the work and resource load on hospitals and staff. While intensifying pharmacological treatment may be considered, our study suggests that two behavioral factors should not be overlooked. Weight reduction and enhancement of medication adherence remains an important mainstream treatment strategy which will help conserve resources in the long term.

## Supplementary information

**Additional file 1.**

## Data Availability

The dataset used and analysed during the current study are available from the corresponding author on reasonable request.

## Abbreviations

ACC: American College of Cardiology
AHA: American Heart Association
ANOVA: Analysis of variance
BMI: Body mass index
BP: Blood pressure
CV: Cardiovascular
CVD: Cardiovascular diseases
ESC/ESH: European Society of Cardiology/European Society of Hypertension
FDC: Fixed-dose combination
HT: Hypertension
MGL: Morisky Green Levine Test
SPRINT: Systolic Blood Pressure Intervention Trial
WHO: The World Health Organization
