# Supplementary material for: Lowered blood pressure targets identify new, uncontrolled hypertensive cases: patient characteristics and implications for services in Thailand
Source: BMC Health Serv Res. 2020 Sep 14;20:869. doi: 10.1186/s12913-020-05719-z (PMC7490895; doi:10.1186/s12913-020-05719-z)
Supplement: Supplementary file 1 — Additional file 1. [file 12913_2020_5719_MOESM1_ESM.pdf]

Study code ________________ DATE _________

1. Demographic data

1.1 Gender ☐ 0.Male ☐ 1.Female

1.2 Age __________ years old

1.3 Height ____________ cm Weight __________kg

1.4 Marital Status ☐ 0.Single ☐ 1.Married ☐ 2.Divorced

1.5 Nationality ☐ 0.Thai ☐ 1.Other ______________

1.6 Religion ☐ 0.Buddhism ☐ 1.Other _________

1.7 Educational level ☐ 0.Primary school ☐ 1. Secondary school ☐ 2. Voluntary school ☐ 3. Bachelor's degree or higher ☐ 4. Other_____________

1.8 Health insurance ☐ 0. Yes ☐ 1. No

1.9 Occupation ☐ 0. None ☐ 1. Government employee ☐ 2. Private company employee ☐ 3.Other ___________

1.9 Involvement of caregiver in medication management

☐ 0.None ☐ 1.Yes Relation________

1.10 Family history of hypertension

☐ 0.None ☐ 1.Yes Relation________

1.11 Cerebrovascular/Cardiovascular disease in family

☐ 0.None ☐ 1.Yes Relation________

2. Health information

2.1 Another health issues ☐ 0. None ☐ 1. Diabetes mellitus ☐ 2. Hyperlipidemia ☐ 3. Other _____________

3. Health behaviors

3.1 Have you exercised (Ex. Power walking, swimming, cycling, aerobic dancing) more than 150 minutes/week in the past 3 months?

☐ 0.No ☐ 1. Yes

3.2 Have you add sodium to your food (fish sauce, soy sauce, tomato/chili sauce)?

☐ 0. No ☐ 1. Add $\geq$1 meal/day

3.3 Have you drunk alcohol in the past 3 months?

☐ 0.No ☐ 1.Yes

3.4 Have you smoked in the past 3 months?

☐ 0.No ☐ 1.Yes

4. Antihypertensive drugs usage

4.1 Number of years diagnosed with hypertension ______________

4.2 Number of years taking anti-hypertensive drugs ______________

4.3 Class of drugs currently taken (review from medical record)

☐ 0. ACEI ☐ 1. ARB ☐ 2. CCB ☐ 3.BB ☐ 4. Other_____________________________

4.4 Types of medication taken per day ___________

4.5 Number of times taken per day ___________

4.6 Any combination drug used? ☐ 0. No ☐ 1.Yes

4.7 Have you experienced antihypertensive drug's side effect? ☐ 0.No ☐ 1.Yes symptom _________

5. Morisky Green Levine Test (MGL)

	**Yes**	**No**
1. Do you ever forget to take your medicine?		
2. Are you careless at times about taking your medicine?		
3. When you feel better do you sometimes stop taking your medicine?		
4. Sometimes if you feel worse, when you take the medicine, do you stop taking it?		
